# Efficacy and Safety of Vitamin E in Adults With Metabolic Dysfunction-Associated Steatohepatitis: A Systematic Review and Meta-Analysis of Randomized Controlled Trials

**DOI:** 10.7759/cureus.88949

**Published:** 2025-07-29

**Authors:** Helai Hussaini, Manpreet Kaur Dhanjal, Rahman Hameed Mohammed Abdul, Olaniyi Fadeyi, Najeeha A Bhatti, Mohammed Qasim Rauf, Calvin R Wei, Adil Amin

**Affiliations:** 1 Internal Medicine, West Anaheim Medical Center, Anaheim, USA; 2 Medicine, Adesh Institute of Medical Sciences and Research, Bathinda, IND; 3 Gastroenterology and Hepatology, Royal Derby Hospital, Derby, GBR; 4 Medicine, Rawalpindi Medical University, Rawalpindi, PAK; 5 Trauma and Orthopaedics, The Hillingdon Hospitals National Health Service (NHS) Foundation Trust, London, GBR; 6 Research and Development, Shing Huei Group, Taipei, TWN; 7 Cardiology, Pakistan Navy Station (PNS) Shifa, Karachi, PAK

**Keywords:** fibrosis, mash, meta-analysis, nonalcoholic steatohepatitis, vitamin e

## Abstract

This systematic review and meta-analysis evaluated the efficacy and safety of vitamin E supplementation in adults with metabolic dysfunction-associated steatohepatitis (MASH), formerly known as nonalcoholic steatohepatitis (NASH). A comprehensive search of PubMed, Cochrane Library, Embase, and Scopus databases was conducted from inception to May 25, 2025, identifying randomized controlled trials comparing vitamin E versus placebo in MASH patients. After screening 752 records, three high-quality randomized controlled trials were included in the final analysis. The pooled analysis demonstrated that vitamin E significantly reduced serum alanine aminotransferase levels compared to placebo (mean difference (MD): -12.27, 95% confidence interval (CI): -16.66 to -7.89) and aspartate aminotransferase levels (MD: -7.08; 95% CI: -14.93 to 0.76). Vitamin E was associated with significantly higher odds of fibrosis improvement (odds ratio (OR): 1.96, 95% CI: 1.25-3.09) with no heterogeneity observed across studies. However, MASH resolution showed no statistically significant difference between groups (OR: 1.71, 95% CI: 0.69-4.27) with substantial heterogeneity, though sensitivity analysis excluding one study revealed a significant benefit. The studies varied in vitamin E dosing from 300 to 800 mg daily, with two conducted in the United States and one in China. These findings suggest that vitamin E supplementation provides biochemical and histological benefits in MASH patients, particularly in reducing liver enzyme levels and improving fibrosis. However, the limited number of trials and varying outcome definitions highlight the need for larger, standardized multinational studies to establish optimal dosing recommendations and long-term safety profiles.

## Introduction and background

Metabolic dysfunction-associated steatohepatitis (MASH), formerly known as nonalcoholic steatohepatitis (NASH), is a progressive form of metabolic dysfunction-associated steatotic liver disease (MASLD) characterized by hepatic steatosis, inflammation, hepatocellular ballooning, and varying degrees of fibrosis [1]. MASH has emerged as a leading cause of chronic liver disease worldwide, largely driven by the global epidemics of obesity, insulin resistance, and type 2 diabetes mellitus [2]. If left untreated, MASH can progress to advanced fibrosis, cirrhosis, hepatocellular carcinoma, and liver-related mortality [3]. Despite the rising burden of MASH and the recent Food and Drug Administration (FDA) approval of resmetirom in March 2024 as the first specific therapy, the need for additional effective and accessible treatment options remains a high-priority area of research [4].

Oxidative stress plays a central role in the pathogenesis of MASH by promoting hepatocyte injury, inflammation, and fibrogenesis [5,6]. Vitamin E, a potent fat-soluble antioxidant, has been proposed as a therapeutic agent for MASH due to its ability to mitigate oxidative stress and lipid peroxidation within hepatocytes [7]. Vitamin E exists in eight different forms, including four tocopherols and four tocotrienols, distinguished by variations in the methyl groups on their chromanol rings and differences in their side chains. The antioxidant properties of vitamin E are primarily attributed to the chromanol head, while the remaining structure influences its biological activity [8]. Over the past decade, several trials of vitamin E at varying doses have been conducted in adults with MASLD, demonstrating positive effects on liver function and histologic parameters [9].

This systematic review and meta-analysis aim to evaluate the efficacy and safety of vitamin E in adult patients with MASH, based on data from randomized controlled trials. Specifically, we assess its impact on key histological endpoints, including resolution of steatohepatitis, improvement in fibrosis stage, and changes in liver enzyme levels, as well as its safety profile. By synthesizing evidence from rigorously designed studies, this review seeks to inform clinical decision-making and clarify the potential role of vitamin E - particularly at low doses - in the management of MASH. Additionally, our analysis aims to address the existing gaps in the literature and guide future research directions in this rapidly evolving field.

## Review

Methodology 

Information Sources 

A comprehensive search was conducted across PubMed, the Cochrane Library, Embase, and Scopus from the inception of each database to May 25, 2025. Key search terms included "vitamin E," "alpha-tocopherol," and "tocopherol" combined with condition-specific terms such as "MASH," "MASLD," "NASH," "NAFLD," "nonalcoholic steatohepatitis," "metabolic dysfunction-associated steatohepatitis," "nonalcoholic fatty liver disease," and "metabolic dysfunction-associated steatotic liver disease." These were further combined with study design terms including "randomized controlled trial," "randomised controlled trial," "RCT," "clinical trial," and "controlled clinical trial." The corresponding Medical Subject Heading (MeSH) terms utilized included Vitamin E [MeSH], alpha-Tocopherol [MeSH], Non-alcoholic Fatty Liver Disease [MeSH]. These terms were combined using Boolean operators. Articles were selected regardless of publication language. Additionally, the reference lists of included studies were manually screened to identify any other relevant studies. The search was performed independently by two authors. Any disagreements were resolved through consensus or consultation with a third author.

Eligibility Criteria 

This systematic review and meta-analysis included only randomized controlled trials (RCTs), irrespective of sample size, that evaluated the effects of vitamin E compared to placebo in individuals diagnosed with MASH. Studies were excluded if they involved children or adolescents (aged <18 years), pregnant women, or individuals with co-existing liver diseases such as alcohol-related liver disease, autoimmune hepatitis, drug-induced liver injury, hereditary liver disorders, or viral hepatitis. We also excluded studies that included participants with secondary causes of liver disease, such as those resulting from total parenteral nutrition or bariatric surgery. In addition, non-randomized studies, reviews, case reports, case series, and animal studies were excluded.

Study Selection and Data Extraction 

Two independent reviewers performed title/abstract screening and full-text assessments using EndNote X9.3.3 (Clarivate, London) for duplicate removal, with discrepancies resolved through consensus or third-reviewer arbitration. Data extraction captured study design characteristics, participant demographics, intervention details (dose, duration), histological outcomes (NAFLD (nonalcoholic fatty liver disease) Activity Score (NAS) score components, fibrosis stage), biochemical parameters (alanine aminotransferase (ALT)/aspartate aminotransferase (AST)), and adverse events, utilizing a standardized piloted form to ensure consistency. 

Quality Assessment 

Methodological quality was assessed using the Cochrane Risk of Bias Tool 2.0 (a framework used to assess the risk of bias in randomized trials), evaluating randomization adequacy, allocation concealment, blinding integrity, outcome reporting completeness, and attrition rates. Studies were classified as low, moderate, or high risk. Quality was assessed by two authors independently. Any disagreement between two authors was resolved through consensus or third-reviewer arbitration.

Statistical Analysis

All statistical analyses were performed using Review Manager (RevMan) version 5.4 software (The Cochrane Collaboration, London). For continuous outcomes, including changes in ALT and AST levels from baseline, mean differences (MD) or standardized mean differences (SMD) with 95% confidence intervals (CI) were calculated using a random-effects model. For dichotomous outcomes including fibrosis improvement and MASH resolution, odds ratios (OR) with 95% CI were computed using the Mantel-Haenszel method. Statistical heterogeneity between studies was assessed using the I² statistic, with values of 25%, 50%, and 75% representing low, moderate, and high heterogeneity, respectively. A p-value <0.05 was considered statistically significant for overall effect estimates. Sensitivity analyses were conducted by systematically excluding individual studies to assess the robustness of pooled results and explore sources of heterogeneity. Given the limited number of included studies (n=3), formal assessment of publication bias using funnel plots or Egger's test was not feasible. All analyses were conducted according to the Preferred Reporting Items for Systematic Reviews and Meta-Analyses (PRISMA) guidelines.

Results 

**Figure 1:**
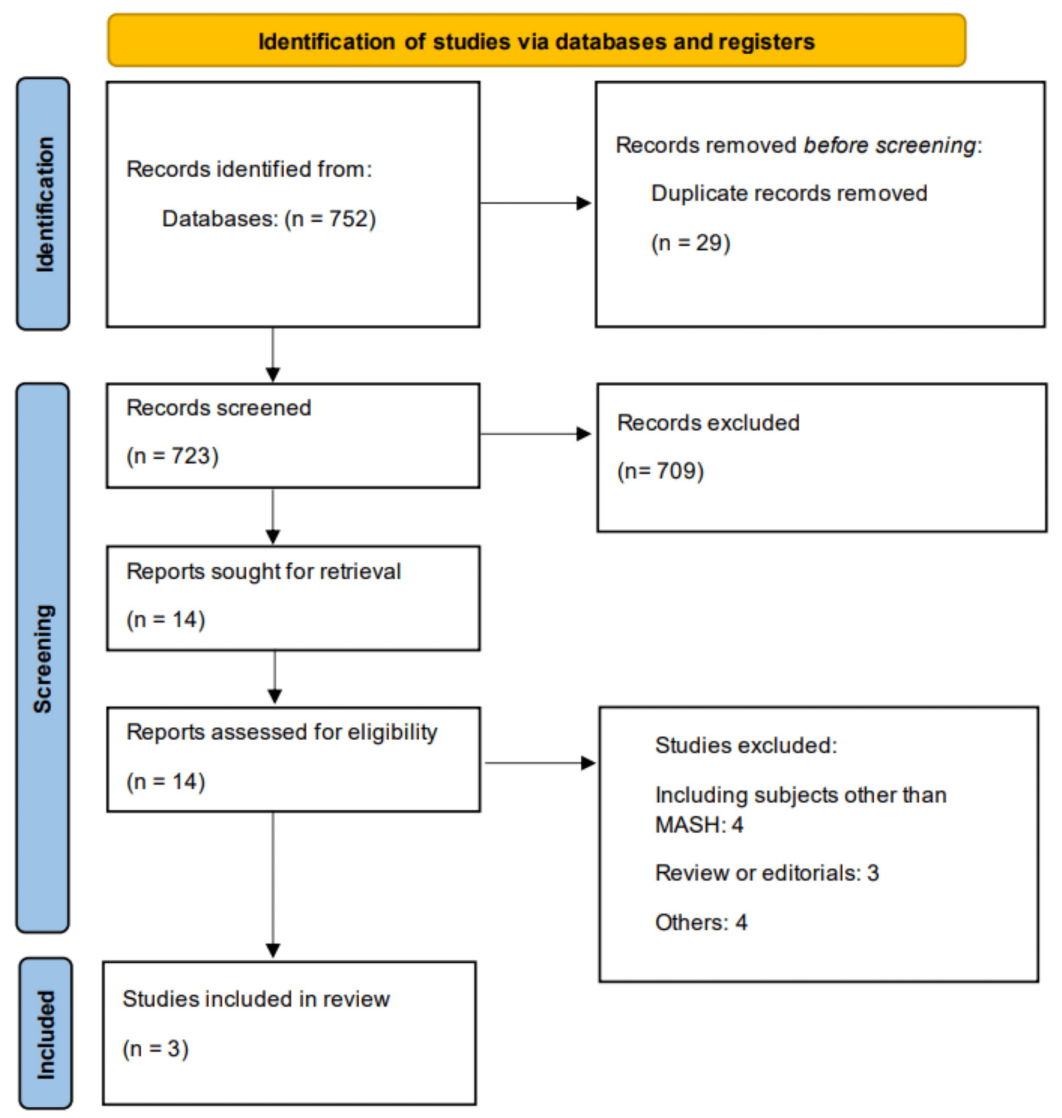
Preferred Reporting Items for Systematic Reviews and Meta-Analyses (PRISMA) flowchart (study selection process)

**Figure 2:**
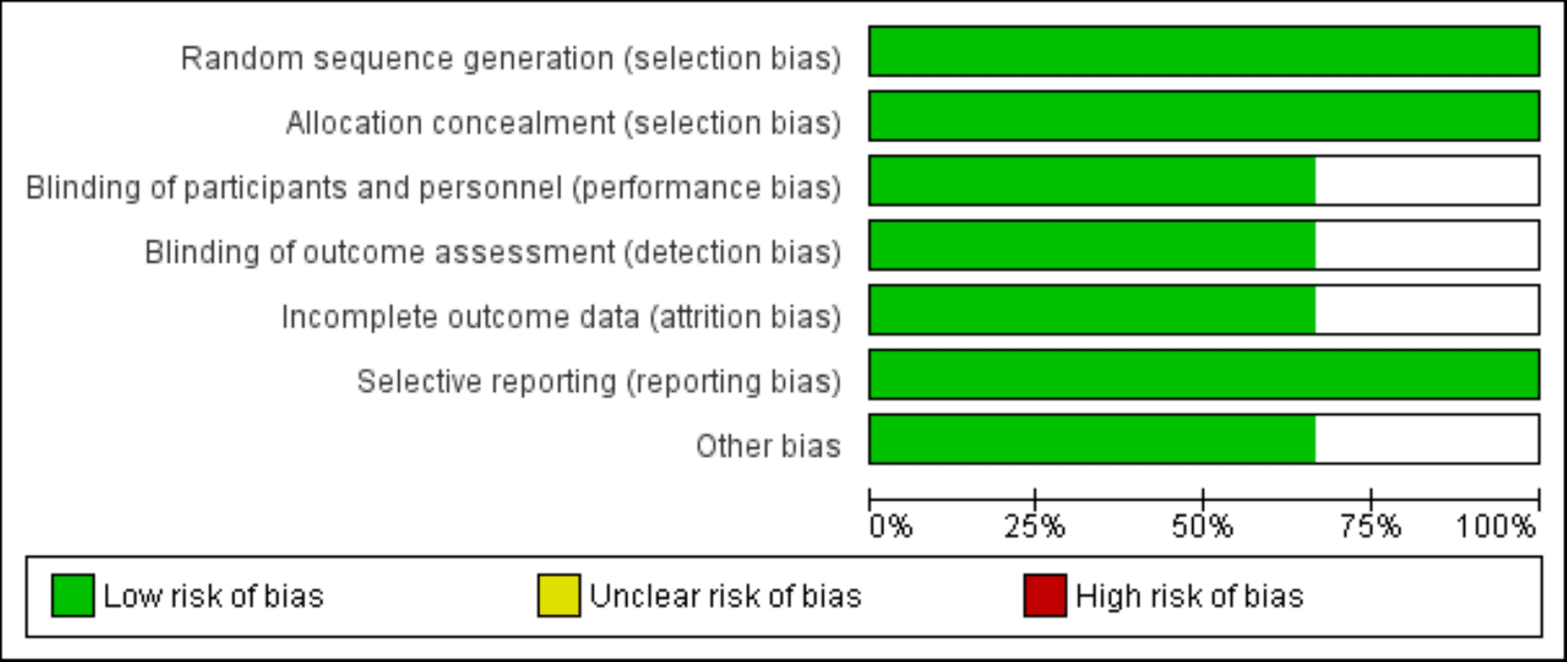
Risk of bias graph

The electronic database search yielded a total of 752 records. From 723 records that remained after duplicates were removed, 709 records were excluded based on title and abstract review. The remaining 14 records were sought for retrieval where 11 records were excluded. Finally, three RCTs were included in this meta-analysis. Figure 1 shows the PRISMA flowchart of study selection. Table 1 presents characteristics of included studies. Two studies were conducted in United States, and one was conducted in China. Figure 2 presents the quality assessment of included studies.

**Table 1:** Included studies characteristics

Author	Year	Region	Groups	Sample Size	Dose of Vitamin E	Follow-up	Mean Age (years)	Males (n)
Bril et al. [10]	2019	United States	Vitamin E	36	800 mg	18 Weeks	60	35
			Placebo	32			57	32
Sanyal et al. [11]	2010	United States	Vitamin E	84	800 mg	96 Weeks	46.6	33
			Placebo	83			45.4	30
Song et al. [12]	2025	China	Vitamin E	58	300 mg	96 Weeks	37.9	42
			Placebo	66			39	50

Changes in ALT from Baseline 

**Figure 3:**
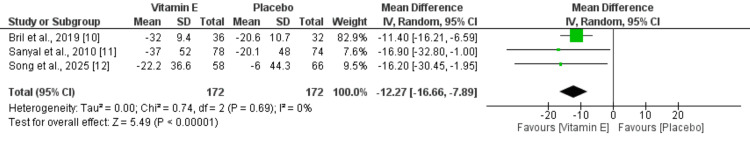
Changes in ALT from Baseline From Refs. [10-12].

Three studies were included to compare the change in ALT levels from the baseline. Based on pooled analysis of three studies, reduction in ALT levels from baseline was significantly greater in subjects receiving vitamin E compared to subjects in the placebo group (MD: -12.27, 95% CI: -16.66 to -7.89) as shown in Figure 3. No heterogeneity was reported among the study results. The study performed by Bril et al. carried higher weight. We performed sensitivity analysis by removing this study. Sensitivity analysis showed similar results with no heterogeneity (MD: -16.51, 95% CI: -27.12 to -5.90, I-square: 0%). 

*Changes in AST from Baseline* 

**Figure 4:**
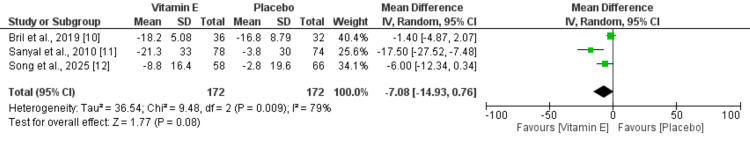
Changes in AST from Baseline From Refs. [10-12].

Three studies were included to compare changes in AST levels from the baseline. The pooled analysis showed a greater reduction in AST levels among participants receiving vitamin E compared to those in the placebo group (MD: -7.08; 95% CI: -14.93 to 0.76), as shown in Figure 4. High heterogeneity was observed across the studies (I² = 79%).

Fibrosis Improvement 

**Figure 5:**
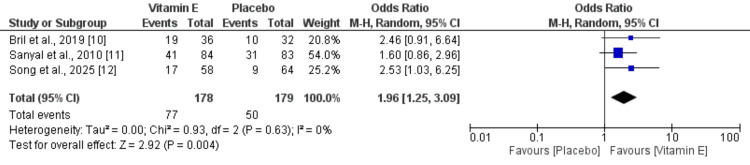
Comparison of fibrosis improvement between two groups From Refs. [10-12].

Three studies were included to evaluate the effect of vitamin E on fibrosis improvement, with pooled analysis results presented in Figure 5. Compared to placebo, vitamin E was associated with significantly higher odds of fibrosis improvement (OR: 1.96; 95% CI: 1.25-3.09). No heterogeneity was observed among the included studies (I² = 0%). 

MASH Resolution 

**Figure 6:**
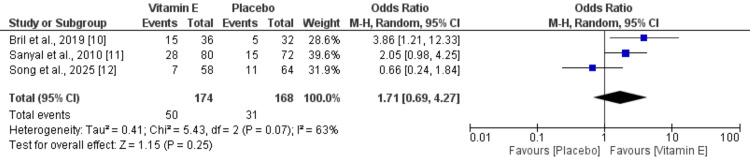
Comparison of MASH resolution between two groups From Refs. [10-12].

Three studies were included to assess the effect of vitamin E on MASH resolution, with pooled results presented in Figure 6. The analysis showed no statistically significant difference between the vitamin E and control groups (OR: 1.71; 95% CI: 0.69-4.27), accompanied by substantial heterogeneity (I²=63%). To explore this heterogeneity, a sensitivity analysis was conducted by excluding the study by Song et al. This analysis revealed a significantly higher likelihood of MASH resolution in the vitamin E group, with heterogeneity reduced to 0% (OR: 2.45; 95% CI: 1.32-4.55).

Discussion 

Our meta-analysis found that vitamin E significantly reduced serum ALT levels and improved fibrosis in individuals with MASH. Our findings also showed a greater reduction of AST levels. These findings are consistent with previous meta-analyses [4,13]. Unlike earlier reviews, our study focused exclusively on patients with MASH and included recently published clinical trials. A recent trial by Song et al. reported a non-significant trend toward higher odds of MASH resolution in the placebo group, which contrasts with the majority of studies included in our meta-analysis. This discrepancy may be attributed to differences in vitamin E dosing - Song et al. used 300 mg daily, whereas other trials employed higher doses (e.g., 800 mg). However, due to the limited number of available studies, we were unable to conduct a subgroup analysis based on vitamin E dosage.

High-dose vitamin E has demonstrated therapeutic benefits in individuals with MASH. In a prospective clinical trial by Harrison et al. [14], daily supplementation with 1,000 IU of vitamin E combined with 1,000 mg of vitamin C over a six-month period led to a significant reduction in liver fibrosis scores confirmed by biopsy, although it did not result in notable improvements in liver inflammation.

The pooled analysis showed a significant reduction in ALT and AST levels among subjects receiving vitamin E. Elevated serum aminotransferase levels reflect hepatocyte membrane damage and inflammation, which are hallmarks of MASH [15]. Sustained reductions in these enzymes are correlated with histological improvements in steatosis, lobular inflammation, and ballooning [4]. For instance, 80% of individuals with MASH who achieved a ≥30% reduction in serum ALT to ≤40 U/L demonstrated histological improvement. Similarly, in the Farnesoid X receptor ligand obeticholic acid in NASH treatment (FLINT) trial, a decrease in ALT of ≥17 U/L was significantly associated with a positive histologic response to obeticholic acid therapy [16]. Although histological outcomes remain the gold standard in MASH drug trials, non-invasive markers such as serum ALT levels are increasingly relevant in clinical practice due to the limitations of liver biopsy, including invasiveness, complication risk, and sampling variability [17].

Vitamin E is believed to reduce ALT and AST levels in MASH primarily through its antioxidant properties. It mitigates oxidative stress by neutralizing free radicals and interrupting lipid peroxidation chains that cause direct damage to hepatocyte membranes [18]. By reducing reactive oxygen species (ROS), vitamin E also exerts anti-inflammatory effects by downregulating pro-inflammatory cytokines such as TNF-α and inhibiting NF-κB pathways, thereby attenuating lobular inflammation [19]. Additionally, it stabilizes hepatocyte membranes by incorporating into lipid bilayers, preventing oxidative disruption and limiting the leakage of intracellular enzymes like ALT and AST into the bloodstream [10]. These combined mechanisms - including oxidative stress inhibition, anti-inflammatory activity, and membrane stabilization - synergistically reduce hepatocellular injury, as reflected by histological improvements in steatosis and ballooning in clinical trials [13]. Our meta-analysis further demonstrated that vitamin E improved fibrosis, a key histological feature. The significant reduction in serum aminotransferase levels supports these histological findings.

One important limitation when interpreting our results alongside previous studies is the lack of standardized definitions for MASH resolution across trials. While most major vitamin E trials required 'no worsening of fibrosis' as part of the resolution criteria, the specific histological definitions varied in both detail and stringency. This lack of consistency represents a broader methodological challenge in MASH research. Our contrasting findings with earlier studies may partially reflect these discrepancies rather than true differences in treatment efficacy. This highlights the need for harmonized histological endpoints in MASH clinical trials, as emphasized in recent regulatory guidance.

Our review also revealed substantial variability in the daily doses of vitamin E used across trials, ranging from 300 to 800 IU. Notably, two prominent international guidelines recommend a daily dose of 800 IU for patients with MASLD [20]. While one meta-analysis reported a 22% increased risk of hemorrhagic stroke associated with vitamin E supplementation [21], another meta-analysis by Alkhenizan et al. found a significant reduction in prostate cancer incidence with its use [22].

Vitamin E was generally well-tolerated in both diabetic and non-diabetic NASH patients, with no significant increase in serious adverse events compared to placebo. However, cardiovascular safety concerns emerged, with four cardiovascular deaths reported in the diabetic study (two in the vitamin E alone group) and similar cardiovascular event rates across treatment groups in the PIVENS trial. Additionally, vitamin E treatment was associated with new-onset diabetes in four non-diabetic patients versus none in the placebo group, though this difference was not statistically significant. These safety findings, while based on studies not powered for safety endpoints, warrant careful cardiovascular risk assessment and monitoring when considering vitamin E therapy for NASH patients.

Study limitations 

Several limitations should be considered when interpreting the findings of this meta-analysis. First, the analysis was based on only three randomized controlled trials, which limits the statistical power and generalizability of our conclusions. The relatively small number of studies also precluded comprehensive sensitivity analyses and robust assessment of publication bias. Second, the lack of individual patient data prevented us from conducting meaningful subgroup analyses based on important clinical variables such as baseline fibrosis stage, diabetes status, body mass index, or demographic characteristics, which may significantly influence treatment response to vitamin E therapy. Third, there was substantial regional variation among the included studies, with one trial conducted in China and two in the United States, potentially limiting the generalizability of findings across different ethnic populations, healthcare systems, and genetic backgrounds. Fourth, the studies employed varying definitions for MASH resolution, with different levels of specificity in histological scoring criteria, which may have contributed to heterogeneity in outcomes and complicated direct comparisons. Fifth, the vitamin E dosing regimens differed substantially across studies (300 mg daily in Song et al. versus 800 mg/800 IU daily in the other two studies), making it difficult to establish optimal dosing recommendations. Sixth, the study populations varied considerably, including non-diabetic patients in two studies and diabetic patients in one study, with different baseline characteristics and disease severity. These limitations underscore the need for larger, standardized, multinational trials with harmonized outcome definitions and longer follow-up periods to better establish the role of vitamin E in MASH treatment. 

## Conclusions

This systematic review and meta-analysis of three randomized controlled trials demonstrates that vitamin E supplementation provides significant biochemical and histological benefits in adults with MASH. Vitamin E significantly reduced serum ALT and AST levels and improved fibrosis compared to placebo, supporting its potential therapeutic role in MASH management. However, evidence for MASH resolution remains inconclusive due to heterogeneity across studies and varying outcome definitions. The limited number of available trials, differences in vitamin E dosing regimens (300-800 mg daily), and varying patient populations restrict the generalizability of these findings. Future research should focus on conducting larger, standardized multinational trials with harmonized histological endpoints, standardized dosing protocols, and longer follow-up periods to establish optimal treatment recommendations and comprehensive safety profiles for vitamin E therapy in MASH patients.
